# Zoonoses As Ecological Entities: A Case Review of Plague

**DOI:** 10.1371/journal.pntd.0004949

**Published:** 2016-10-06

**Authors:** Caio Graco Zeppelini, Alzira Maria Paiva de Almeida, Pedro Cordeiro-Estrela

**Affiliations:** 1 Programa de Pós-Graduação em Ciências Biológicas, Centro de Ciências Exatas e da Natureza, Universidade Federal da Paraíba, Campus I, João Pessoa, Paraíba, Brazil; 2 Laboratório de Mamíferos, Departamento de Sistemática e Ecologia, Centro de Ciências Exatas e da Natureza, Universidade Federal da Paraíba, Campus I, João Pessoa, Paraíba, Brazil; 3 Centro de Pesquisa Aggeu Magalhães Fiocruz, Campus da Universidade Federal de Pernambuco, Recife, Pernambuco, Brazil; UTMB, UNITED STATES

## Abstract

As a zoonosis, Plague is also an ecological entity, a complex system of ecological interactions between the pathogen, the hosts, and the spatiotemporal variations of its ecosystems. Five reservoir system models have been proposed: (i) assemblages of small mammals with different levels of susceptibility and roles in the maintenance and amplification of the cycle; (ii) species-specific chronic infection models; (ii) flea vectors as the true reservoirs; (iii) Telluric Plague, and (iv) a metapopulation arrangement for species with a discrete spatial organization, following a source-sink dynamic of extinction and recolonization with naïve potential hosts. The diversity of the community that harbors the reservoir system affects the transmission cycle by predation, competition, and dilution effect. Plague has notable environmental constraints, depending on altitude (500+ meters), warm and dry climates, and conditions for high productivity events for expansion of the transmission cycle. Human impacts are altering Plague dynamics by altering landscape and the faunal composition of the foci and adjacent areas, usually increasing the presence and number of human cases and outbreaks. Climatic change is also affecting the range of its occurrence. In the current transitional state of zoonosis as a whole, Plague is at risk of becoming a public health problem in poor countries where ecosystem erosion, anthropic invasion of new areas, and climate change increase the contact of the population with reservoir systems, giving new urgency for ecologic research that further details its maintenance in the wild, the spillover events, and how it links to human cases.

## Introduction

The world experiences a new epidemiologic transition where the scenario has been profoundly changed by globalization and anthropic impacts in the biosphere, creating a window for the emergence and rapid dissemination of new infections and rekindling diseases that were considered under control [1]. In the specific case of zoonosis, much attention is given to outbreak dynamics, vector control, and vaccine development, which is most understandably an anthropocentric viewpoint. However, if we want to be able to develop predictive models or build alert systems, we must increase focus on zoonoses as ecological entities. This task alone is daunting because of the huge diversity of aetiological agents, hosts, interactions, and abiotic dynamics. As most generalizations might be only truisms, we focused our discussion of this topic on Plague because of its remarkable historical impact on human history that contrasts with its current status and understanding. We hope that our analytic framework for Plague could be applied to other zoonoses that have multiple hosts (e.g., Lyme disease, Leishmaniasis). In the present paper, we highlight biases in research and surveillance activity, emphasize the main role of the reservoir system, and stress the need to include anthropic landscape changes into predictive models. Here, Plague poses both as a problem to be understood and solved and as a model to comprehend the zoonosis ecology approach.

Plague is a disease caused by *Yersinia pestis*. It primarily affects rodents, but other mammals including cats, dogs, rabbits, camels, and humans can be infected [2]. Old World rats conveyed the bacteria throughout the world with transmission operating through their fleas; with the spreading of the disease across the globe, diverse sylvatic reservoir systems evolved by an assembly of hosts with different susceptibilities, assuring permanent bacterial circulation [3]. Little is known about the ecology of sylvatic Plague, because the surveillance efforts focus solely on collecting data that indicates outbreak risk for public health measures [4]. Furthermore, studies concentrate on few foci (mostly North American foci, that account for 1.2% of the recent human cases [5]), with focal approaches, leaving several questions, such as global patterns and restricted spatial occurrence, mostly unanswered. In fact, the ecology (at autoecological and/or community level) has not been thoroughly analysed to examine which conditions favour the maintenance of *Y*. *pestis* in sylvatic systems. Whole continents—like South America—lack proper ecological studies of their foci [6], hindering any analysis on the common mechanisms and characteristics that underlie the Plague dynamics worldwide. Issues like its maintenance in time and space (including the quiescence periods), the role of the mammal and flea faunas in the enzootic/epizootic cycles, and the mechanisms underlying the passage from maintenance cycles to outbreaks remain poorly understood. The lack of clear environmental determinants of Plague occurrence suggest that interactions between factors might promote conditions for the emergence of Plague outbreaks rather than factor themselves. Although several reviews on Plague were published in the last decade (e.g., Bevins et al. [7]), little attention has been paid to the role of the community structure on its ecology, an understudied aspect on pathogen ecology [8].

## A Community Approach: The Reservoir System

Primarily, Plague is a parasitic relationship between bacteria (*Y*. *pestis*) and hosts, as well as a parasitic relation between the vector (fleas) and the hosts. Parasitism is an ecological relation that involves at least two species (parasite and host), but normally involves an array of host species, vectors and species that have direct and indirect links to the host species [8], constituting one of the most common ecological interactions known and the most common feeding strategy in nature [9]. Plague is a zoonotic disease, where the host community is known as a reservoir system (sensu Roche et al. [10]). Plague’s reservoir systems across the foci are mainly composed of rodents, but with relatives of other mammal taxa participating in the cycle, such as Didelphimorphia marsupials [11] and carnivores (canids, felids, mustelids) [7]. Humans configure incidental hosts, as they develop high bacteraemia (septicaemia) very quickly and tend to die within a very narrow time frame [2].

The role of rodents as Plague reservoirs is fairly well understood, as empirical data show that they are the organisms most commonly infected and that their fleas are the competent vectors. Yet, this might represent a strong research bias developed since the role of rodents and fleas was determined [2], directing efforts towards this taxa pair. Plague has been detected in groups as diverse as camels (Cetartiodactyla) and humans (Primates), and over 200 mammal species have had confirmed infection by *Y*. *pestis* [7], possibly indicating that the role of reservoirs is likely not restricted to rodents and/or that field research neglects a substantial part of potential reservoir systems, leading to inaccurate conclusions about the sylvatic cycle of infection.

**Fig 1:**
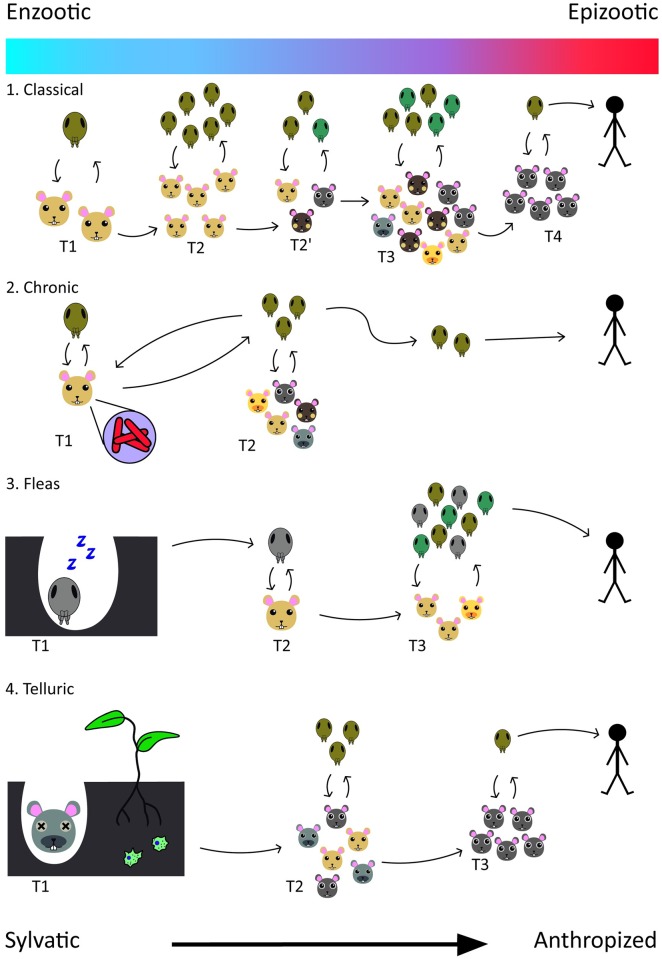
The four reservoir models proposed by Poland and Barnes [12], revisited by Gage and Kosoy [13]. From the top down: 1. classical model, 2. chronic infection model, 3. fleas as reservoir, 4. Telluric Plague. Horizontal axis informs the spatial occurence of the phenomena, starting from a sylvatic landscape to a more urban/periurban situation, indicating public health risk. The gradient bar indicates the possibility of transition from an enzootic to an epizootic cycle. Rodent species differentiated by color and pattern, flea species by color alone. Arrows indicate the interaction between the components of the cycle in given time T (double arrows), or the progression of each stage of the model. Arrows pointing towards humans indicate human infection in epizootics.

There are four sylvatic reservoir models proposed for Plague [12,13] (Fig 1). The first hypothesis comes from the traditional idea of rodent assemblage being the wild reservoir of Plague, stressing the necessity of an assemblage that is active throughout the year to keep a steady transmission cycle (hibernation would force the cycle into a halt). The second hypothesis claims species or populations capable of developing a chronic infection—even presenting granuloma-like lesions infested by *Y*. *pestis*—and infect vectors continuously as reservoirs. The third hypothesis places fleas as a true reservoir system, not just vectors, with empirical data proving that several species of fleas survive up to 558 days with a complete blockage and no feeding. Following a period of suspended activity or latency inside burrows, the fleas could restart their feeding activities and contaminate rodents to rekindle the transmission cycle. The fourth hypothesis points toward the survival of *Y*. *pestis* contaminating soil, parasitizing soil Protozoa and plant tissues, and/or in nonculturable state.

**Fig 2:**
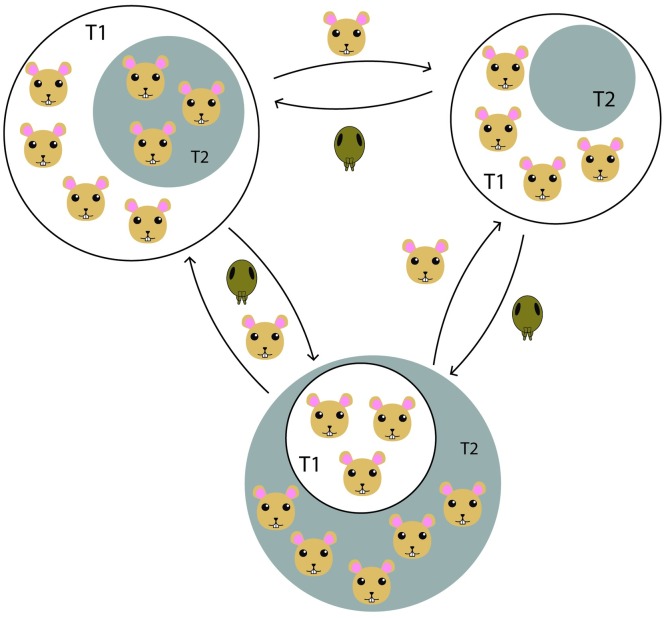
The metapopulation model for Plague reservoir systems. Discrete subpopulations are disjoint in space but maintain contact through movement of individuals. Population size fluctuations are indicated by the white circle (T1, first assessment) and the gray circle (T2, second assessment), where populations can retract, expand, or extinguish. Arrows indicate movements of individuals (rodents or fleas) that maintain the subpopulations interconnected and working as a functional unit. For detailed information on the effects of population fluctuations on Plague cycles, see Reijniers et al. [15, 16].

Yet, a viable (and active) model for the sylvatic maintenance of Plague is overlooked by the authors: the metapopulation arrangement (Fig 2). It has been detected and characterized in foci of the western United States [14], Kazakhstan [15,16], and (putatively) Madagascar [5]. Commonly, the species that form “metapopulation reservoirs” are very susceptible and tend to have high die-off rates [7,14]. The metapopulation arrangement functions as a spatial relation, where the subpopulations have some degree of isolation from one another, allowing focal outbreaks, affecting one or a subset of subpopulations. After the population depression or decimation, the adjacent subpopulations would be able to recolonize the “cleared” area [17] (similar to the source-sink dynamics). The arrangement is evident in areas where the affected species have a well-delimited area of occupation, forming “burrow cities,” as in the US (where the primary reservoir species are the prairie dogs, *Cynomys* spp.) and Kazakhstan (gerbils, *Rhombomys* spp.), and allows the maintenance and circulation of *Yersinia* without the presence of resistant hosts, probably by the continuous movements between subpopulations [18] with secondary hosts involved during the quiescence periods, whose population threshold marks the risk of epizootic events by percolation, as observed by Salkeld et al. [19]. Community composition and structure can enhance the predictability of enzoosis/epizoosis cycles. Reijniers and colleagues [15,16] discuss the paradigm of abundance thresholds. The pathogen installation in a new environment depends on a minimal host abundance (critical population size), manifested as timespans of optimal population sizes for invasion and/or outbreak; coupled with a density threshold for the vector, referring to a minimal abundance of fleas infecting the naïve hosts, and a minimal infestation of positive hosts to efficiently spread the pathogen throughout the population. The population levels for vector and host species combined define the thresholds, below which the cycle is interrupted. The models projected by Reijniers et al. [15,16] for the foci in Central Asia predicted a two-year delay in the response (outbreaks) for population fluctuations, albeit the authors warn that the observed effects are for detectable infection, and might not fully represent the infection profile per se.

Enzootic hosts are defined as “primary hosts” (i.e., capable of long-term supporting the pathogen in the wild), the “secondary” and “tertiary” hosts would be the more susceptible ones, responsible for amplifying and causing the spillover, initiating an outbreak [20]. Empirically, there is evidence of the participation of wild tertiary and incidental hosts (including carnivores and ungulates) on the epizootic cycle at local scales, through carrying of infected fleas over a broader area than the rodents alone would be able to cover by their landscape movement [12]. According to Gage and Kosoy [3], the enzootic hosts would be resistant to the infection, having low obvious mortality rate, surviving and carrying the infection for long periods of time, in contrast to the epizootic (amplifying) hosts that would have a fast-paced die-off, which constitutes the epizootic event itself. Yet, the authors warn that the separation between enzootic and epizootic events is often dubious, as both are density-dependent phenomena, and events regarded as epizootic could be simply cases of higher mortality among enzootic hosts or a more efficient detection of the natural die-off during the enzootic phase. Such exercise is important to comprehend the nature of reservoir systems, as in some foci a single species is held accountable for maintaining the infection, such as *Rattus rattus* in a Malagasy focus [21] and *Necromys lasiurus* (*Zygodontomys lasiurus pixuma*) in Brazil [22].

If the importance of community structure and composition to the epidemiology of zoonosis is increasingly acknowledged [23], the taxonomy of species of reservoir systems can often be neglected. Although in a substantial part of the world, the small mammal assemblies are composed almost solely by rodents, in Africa and South America there are other small mammal taxa (Soricomorpha and marsupials, respectively) that have received little to no attention on Plague community studies although being known as actual and potential reservoirs for other zoonoses that affect humans [24,25]. This taxonomical bias might result in underestimated reservoir systems and incomplete understanding of the sylvatic cycle. Malek et al. [26] detected *Crocidura russula* positive for *Y*. *pestis* in Algeria, hinting that Soricomorpha might be involved in transmission [27]. As several characteristics of the species, such as life history, variance in susceptibility, and mortality, might play a significant role in plague dynamics, accurate taxonomic identification is of utmost importance. In a recent review of South America, 22 of 50 rodent hosts of *Y*. *pestis* underwent taxonomic changes in the last 20 years [28]. Furthermore, the taxonomic knowledge of the reservoir system affects sampling practices conducted during studies in foci. Most studies happen within human occupation areas, specific time frames (during or after epizootic events) employing methods that enhance the capture of certain taxa or exclude others [27].

Although works cited conducted surveys in forest fragments, the gross majority of samplings were held on anthropized environments (villages, crop fields, residences), filtering the assemblage capable of occupying the area [29] and possibly portraying a subset of the real reservoir system. Knowledge on the actual assemblage demands systematic surveys to avoid biases; and few studies were conducted to achieve this refinement in ecological information. One remarkable example of such is the Plano Piloto da Peste (PPP, Plague Pilot Plan), a research project idealized by Marcel Baltazard from Institut Pasteur of Paris, focused on understanding the assemblage (emphasizing sylvatic rodents) of a focus in northeastern Brazil, the roles of each species, and ecological aspects regarding landscape ecology [30]. The Plague Pilot Plan lasted about a decade and represented the most refined study in Plague ecology and epidemiology in South America, with valuable contributions to understand the roles of hosts and vectors (e.g., Karimi et al. [31]).

It is necessary to understand the reservoir systems’ structure, diversity of networks, and ecosystem context of zoonoses to comprehend their dynamics and the human impact on their current mechanics [10]. The notion that Plague can affect ecological links between members of the reservoir system and higher trophic levels mandates a broader picture of the ecosystem relations between reservoir system and sympatric species [20].

## Environment and Plague

A purely zoological approach falls short from understanding any pathogen transmission cycle throughout time and space. The cycles are ecological interactions between at least two species, but also include their relationship with other species and their environment, a whole ecosystem interaction [10]. The transmission cycle has an ecological “niche” of its own, as a sum-of-the-parts of its agents’ ecological characteristics, and can be analysed through ecological niche modelling (ENM) [32]; which has already been used to some extent on Plague systems [33], assessing the relationship of the transmission cycle with landscape and climate features to which the transmission cycles are strongly linked [34]. Ecosystem approaches evidenced traits such as the foci being present in altitude varying as much as about 500 m in Brazilian foci, and 1,000+ m in Andean and African foci [6,27], the influence of global climatic phenomena (such as El Niño) [35], and the local responses to precipitation variations [33,36,37].

Climatic conditions (precipitation patterns, temperature) play a prominent role in the seasonal fluctuations of Plague and in the outbreak of epizootics. Mild winters and wet climate in the winter–spring interface correlates with increased human and sylvatic cases with a small lag [18,27,37]. Such relation is commonly addressed as an effect of Trophic Cascade [38]. Climate change plays an important role on the current behaviour of zoonoses, as climate itself is a prime factor on transmission cycles [34]. Climate change is expanding the range of vector-borne diseases (Plague included) towards the poles and higher elevations [34], as it makes suitable for the vectors (and in some cases, the hosts) areas that were too cold and/or dry for their survival and establishment, as it is already noticed in California (US) [39].

Plague is associated with human occupation and following landscape management, as occupying former natural habitats is the main way humans get in contact with wildlife diseases [8]. Anthropic landscape alterations usually come as fragmentation, increasing ecotone area and enhancing chances of contact with hosts and vectors of zoonotic pathogens [40]. Plague is more present in rural and/or suburban occupations lacking sanitary infrastructure and low socioeconomic status [38]. Small mammals are attracted to the anthropized areas by available resources (i.e., crops, rubbish) and possible burrow sites, carrying fleas and bacteria, and creating almost direct contact with the sylvatic transmission cycle [41]. The anthropized landscape filters diversity, enhancing risk of transmission if the species that manage to thrive in the altered landscape comprise good enzootic reservoirs and good amplifier hosts [8]. Impoverished diversity enhances contact rates between vectors and competent hosts, increasing chances of transmission, a reverse dilution effect [10]. Conditioning animal movement and dispersal, topography and landscape structure help with understanding the spatial dispersal of Plague, especially for metapopulation. Landscape heterogeneity offers areas with different qualities, acting as pathways or barriers, dictating probable directions and contact rates between subpopulations, especially in cases where hosts form spatially structured distribution (colonies), like prairie dogs and gerbils [17]. Plague has negative association with structures like rivers, canals, and roads that interrupt possible movement of hosts, vectors, and/or predators (which can carry fleas or contaminated carcasses) [5]. Human movements across space also play an important role on Plague dynamics, inadvertently carrying rodents and fleas with their cargo and/or transport patients in contagious phase and disseminate infection [38].

## Future Insights and Perspectives

Although we summarize what is known for Plague in population and spatial and community ecology, fieldwork specifically designed to assess that aspect is essential to provide answers. Three issues need to be studied with real world data and models. It is important to find out to what extent other taxonomical groups have a participation in the cycles; Moore et al. [27] is the first assessment of its kind and concurs with our view that other groups of small mammals intimately linked with rodents—occupiers of the same areas, potentially share parasites and predators, and captured with the same techniques used for rodents—have direct participation in maintaining and/or amplifying Plague in nature. There is great necessity to study Plague out of human occupation spectre, as we consider that different assemblages found in preserved habitat patches might have different roles in its maintenance, possibly answering questions such as maintenance during quiescence periods. There is the need of looking deeper into the ecological relations that surround and affect Plague systems, such as predation and competition, and how they can affect the role of the species involved.

For abiotic variables, the ideal analysis should encompass all landscape and climate features, as they are responsible for driving the ecological relations in an area, creating a comprehensive picture that could not be assessed by purely biological approaches. Yet, such analysis is far from possible because of the amount of variables that need to be added to the equation, restricted data available to support the analysis, and especially the refinement and resolution of data. Plague foci have a strong geographical delimitation, while some of the environmental data available, such as climate models, work in a larger resolution, rendering imprecision to the analysis (e.g., Moore et al. [18]). Despite Plague being far from under control in Africa, it could be one of the “rekindled” infectious diseases, depending on the outcome of increased global connectivity, ecosystem erosion, and biodiversity loss in its cycle.

## Conclusion

Plague is a complex ecological entity, so far not understandable with a single model; its diversity of reservoir models and transmission cycles makes difficult the task of creating a satisfactory general model to fit the plurality observed. Despite the possible resultant bias of detectable infection versus actual infection rates, we believe that community size modelling is a valuable tool to understand the role of the host assemblage as a whole, the individual contribution of each species on the spatial and temporal dynamics of the infection, and demands testing in a broader diversity of Plague systems. The study bias that neglected the understanding of native rodents in the maintenance of sylvatic Plague is already identified as a concerning issue [13].The need for studies encompassing the relationships between populations and landscape, as well as community interactions, are the best way possible to achieve fuller comprehension of the nature of cycles.

**Key Learning Points**

- Plague could be considered an ecological entity characterized primarily by the interaction between host, vectors, and spatiotemporal and environmental variables.
- Environmental impacts and landscape management practices foster contact between humans and *Y*. *pestis* and can increase public health risks.
- Plague presents changes in its behavior and distribution with climate change.

**Top Five Papers**

1. Gage KL, Kosoy MY. Natural history of plague: perspectives from more than a century of research. Annu Rev Entomol. 2005;50: 505–528. doi:10.1146/annurev.ento.50.071803.130337
2. Schneider MC, Najera P, Aldighieri S, Galan DI, Bertherat E, Ruiz A, et al. Where does human plague still persist in Latin America? PLoS Negl Trop Dis. 2014;8: e2680. doi:10.1371/journal.pntd.0002680
3. Keesing F, Belden LK, Daszak P, Dobson A, Harvell CD, Holt RD, et al. Impacts of biodiversity on the emergence and transmission of infectious diseases. Nature. 2010;468: 647–652. doi:10.1038/nature09575
4. Roche B, Rohani P, Dobson AP, Guegan JF. The impact of community organization on vector-borne pathogens. Am Nat. 2013;181: 1–11. doi:10.1086/668591
5. Pongsiri MJ, Roman J, Ezenwa VO, Goldberg TL, Koren HS, Newbold SC, et al. Biodiversity Loss Affects Global Disease Ecology. Bioscience. 2009;59: 945–954. doi:10.1525/bio.2009.59.11.6
